# Biomonitoring along the Tropical Southern Indian Coast with Multiple Biomarkers

**DOI:** 10.1371/journal.pone.0154105

**Published:** 2016-12-12

**Authors:** Sivanandham Vignesh, Hans-Uwe Dahms, Krishnan Muthukumar, Gopalaswamy Vignesh, Rathinam Arthur James

**Affiliations:** 1 Department of Marine Science, Bharathidasan University, Tiruchirappalli, Tamil Nadu, India; 2 Department of Marine Biotechnology and Resources, National Sun Yat-sen University, No. 70, Lienhai Road, Kaohsiung, Taiwan, R.O.C.; NSYSU; 3 Department of Chemistry, Bharathidasan University, Tiruchirappalli, Tamil Nadu, India; Seoul National University, REPUBLIC OF KOREA

## Abstract

We assessed the spatial and temporal variations of pollution indicators and geochemical and trace metal parameters (23 in total) from water and sediment (144 samples) of three different eco-niches (beach, fishing harbor, and estuary) in larger coastal cities of southern India (Cuddalore and Pondicherry) for one year. A total of 120 marine *Pseudomonas* isolates were challenged against different concentrations of copper solutions and 10 different antibiotics in heavy metal and antibiotic resistance approaches, respectively. The study shows that 4.16% of the isolates could survive in 250 mM of copper; 70% were resistant to minimum concentrations. Strains were resistant (98.4%) to at least one antibiotic in Cuddalore compared to the Pondicherry (78.4%) region. Pollution index (PI) (0–14.55) and antibiotic resistance index (ARI) (0.05–0.10) ratio indicated that high bacterial and antibiotic loads were released into the coastal environment. The degree of trace metal contamination in sediments were calculated by enrichment factor (EF), contamination factor (CF), pollution load index (PLI), and geo-accumulation index (*I*_*geo*_). Statistical parameters like two-way analysis of variance (ANOVA), correlation, factor analysis and scatter matrix tools were employed between the 23 parameters in order to find sources, pathways, disparities and interactions of environmental pollutants. It indicates that geochemical and biological parameters were not strongly associated with each other (except a few) and were affected by different sources. Factor analysis elucidated, ‘microbe–metal’ interaction (Factor 1–48.86%), ‘anthropogenic’ factor (Factor 2–13.23%) and ‘*Pseudomonas*–Cadmium’ factor (Factor 3–11.74%), respectively.

## Introduction

Coastal pollution can be caused by activities in large urban centers that have insufficient cleaning and being without sewage and wastewater treatment facilities. This may be particularly problematic during high tourist seasons and mass public visits on weekends because the municipalities are not able to cope with excess refuses that are reaching beaches, coastal lagoons, and the sea, and this way affecting the sanitary conditions [1]. However, in fishing harbors and estuaries, both point and non-point wastes are frequently reaching the coastal system. These wastes as direct human and animal excretions as well as untreated sewage may damage the coastal environment [2]. The build-up of illegal settlements on river banks/ estuaries and several fisher folk houses with improper sanitary facilities additionally contribute to high pollutant levels. Various factors like tidal phenomena, presence and location of sewage outlets, seasonality, presence of animals, and increasing numbers of beachgoers are promoting the survival and dispersion of fecal index microorganisms and pathogens in coastal zones [3,4]. The physical stresses/ action may channelize pollutants to the coastal zone or concentrate wastes in areas for a few days or may accumulate in the sediment column or along the shoreline [5]. Microorganisms play a key role in both water and sediments for nutrient cycling, organic matter diagenesis and energy transfer processes, biogeochemical cycles, and benthic trophodynamics [6,7].

Coastal zones are considered as a major sink for multiple pollutants and might have major ecological effects through their concentration, persistence and bioavailable toxicants, even at trace level concentrations [8]. An uncontrolled, extensive, improper usage and disposal of antibiotics in human/ veterinary medicine, agriculture and aquaculture has created a pressure on enteric and aquatic bacterial communities [2]. Toxic heavy metals, pesticides, hydrocarbons and other contaminants could easily enter into the aquatic system. Trace metal contents in the water and sediment columns can be of natural and of anthropogenic origin and are influenced by weather/ temperature, wave/ tidal action, water current, sediment texture, mineralogical composition, reductive/ oxidative state, adsorption/ desorption and physical transport [9]. Normally, the availability of trace metals in any environment depends on their chemical form and speciation [10]. Trace metals commonly accumulate in aquatic organisms and they can cause significant modifications here, affecting metabolic pathways, enzyme functioning, ion uptake, nutritional uptake, growth rates, and causing mutations [11]. This may enhance their genetic modification and adaptability via mutation and gene transfer to survive unfavorable conditions.

The present study is focusing on the seasonal/ temporal variations of various chemical and biological pollutants with their impacts on the organism nexus to human health in the coastal zone coupled with statistical tools for understanding the geochemical and microbial inter/ intra-relationships.

## Materials and Methods

### Study area

**Fig 1:**
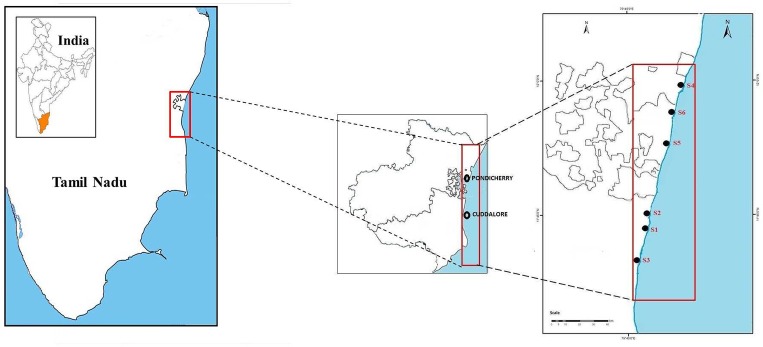
Sampling sites at the Cuddalore and Pondicherry coastal areas.

No specific permissions were required and samplings did not involve endangered or protected species. Cuddalore (latitude 11.75°N and longitude 79.75°E) is a fast growing industrial city and an important coastal city in Tamil Nadu state of southern India. Cuddalore has many small and large industries along the coastline which employs inhabitants of the city. It also receives much anthropogenic pollution and toxic metal/ chemicals from diverse sources from the inland frequently. Pondicherry is a large municipality in the territory of the Indian union, situated between latitude 11.93°N and longitude 79.83°E and is a most popular tourist/ weekend destination in southern India. In recent years, these cities underwent extensive changes due to industrialization, urbanization and increasing population pressure. Our sampling sites were located in areas with different contamination. The sampling seasons fall into four groups: post monsoon (January–February), summer (March—May), premonsoon (June—August), and monsoon (September—December) [1]. The study sites (Fig 1) were as follows: Cuddalore beach (S1), Cuddalore fishing harbor (S2), Cuddalore Uppanar river estuary (S3), Pondicherry beach (S4), Pondicherry fishing harbor (S5) and Pondicherry Sangarabarani river estuary (S6). The sampling locations were demarcated by using a geographical positioning system (GPS) (Garmin GPS 60).

### Sampling

A total of 144 (water and sediment) samples from three different eco-niches in both cities (Cuddalore and Pondicherry) were collected during one year from December 2013 to November 2014 (sampling dates: Postmonsoon = December 24th; January 26th & February 25th; Summer = March 24th; April 25th & May 24th; Premonsoon = June 24th; July 25th & August 25th; Monsoon = September 25th; October 24th & November 25th) for three different analytical approaches (bacteriological, geochemical, and trace metal analysis). Single random sampling was performed at three times during each season. A sample of 2000 ml of marine water was collected from 0–20 cm below the surface with a 2500 ml sterile container at each location. Physicochemical parameters i.e., pH, electrical conductivity (EC), total dissolved solids (TDS), and salinity were measured using a field kit (Thermo Orion 5-Star pH Multi-Meter) at the site and, dissolved oxygen (DO), biological oxygen demand (BOD), total alkalinity (TA) and total hardness (TH) were measured in the laboratory using a standard procedure [12,13]. Approximately 250 g of surface sediment samples were collected (0–5 cm depth) with a sterile spatulum and stored in aseptic polyethylene bags. All samples were kept in iceboxes and processed within 10 h of collection. For heavy metal analysis of water, one liter of sea water was acidified immediately with concentrated nitric acid.

### Bacteriological analysis

Bacterial populations from water and sediment samples were analyzed by the pure culture technique (spread plating method) on specific selective media (S1 Table) plates with 100 μL of suitable serial dilutions [14]. The media plates were incubated at 37 ± 1°C for 24 to 48 h except M-FC plates (M-FC agar plates were incubated at 44.5°C ± 1°C for 24–48 h) when the final counts of colonies were noted. Bacterial colonies from the selective media were considered as similar (“-like”) to known microorganisms (LO) and Rapid Microbial Limit Test kits were used for bacterial identification [3,15]. The pollution index (PI) was calculated using the ratio of fecal coliforms (FC)/ fecal *Streptococci* (FS) [2,16]. All culture media were obtained from Hi-Media Pvt. Ltd., Bombay, India.

### Determination of antibiotic resistance

A total of 120 marine *Pseudomonas* were isolated from water and sediment samples by using selective media (cetrimide agar) plates. Twenty colonies of *Pseudomonas* sp. were isolated from each region and challenged against ten standard antibiotics on Mueller Hinton agar (MHA) by the disc diffusion method for multiple antibiotic resistance analysis [2]. The results were interpreted based on the recommendations of the National Committee for Clinical Laboratory Standards for antimicrobial susceptibility testing [17]. Ten standard antibiotic discs represented seven different chemical structural classes of antibiotics: aminoglycosides (gentamycin 10 mcg), ß-lactams (amoxicillin 10 mcg, ampicillin 10 mcg, methicillin 10 mcg and penicillin-G 10 mcg), glycopeptides (vancomycin 10 mcg), macrolides (erythromycin 10 mcg), quinolones (ciprofloxacin 10 mcg), tetracyclines (tetracycline 10 mcg) and others (chloramphenicol 10 mcg). The antibacterial resistance index (ARI) of each location was calculated according to Hinton et al. [18], using the formula *ARI* = *y* / *nx*, where ‘y’ represents the actual number of resistance determinants recorded from a population of size ‘n’ and ‘x’ as the total number of antibacterials tested in the sensitivity test.

### Assessment of metal toxicity

A total of 120 *Pseudomonas* isolates were challenged against five different concentrations (5, 10, 50, 100 and 250 mM) of copper metal salt solution for metal resistance studies by plate diffusion and tube dilution methods. A stock solution of copper (CuSO_4_.5H_2_0) was prepared in triple distilled water and was sterilized at 121°C for 15 min. For a plate diffusion assay, 500 μL of copper solution was added to a central well (1 cm in diameter and 4 mm in depth) of each nutrient agar plate to allow metal diffusion for one day. Eight isolates were inoculated in each plate by the radial streaking method and were incubated at 37±1°C for 48 h. All the trials were performed in triplicate. After incubation, the percentage of bacterial tolerance was calculated in terms of the ratio: length of growth in mm *vs* length of the total inoculated streak. For the tube dilution method, the appropriate amount of metal solution and 200 μL of standard culture (test culture suspension prepared in sterile 0.85% saline matching an optical density of 0.5 McFarland standards corresponding to 108 CFU/mL) were added into nutrient broth containing test tubes, and the final volume of 10 ml was made-up with sterile nutrient broth. Tubes were read after incubation at 37±1°C for 5 days [19].

### Assessment of heavy metals

One liter of sea water was filtered through a 0.45 μm nitrocellulose membrane filter paper and adjusted to pH 2 with HNO_3_ taken in a separatory funnel. From a freshly prepared solution of amino-pyrolidine dithiocarbamate (APDC) 10 ml (3% w/v) were added into the funnel, and the mixture was shaken by a mechanical shaker for 10 min. Furthermore, 25 ml of methyl-isobutyl-ketone (MIBK) was added to this mixture and shaken for 15 minutes. The phases were allowed to separate and the top organic phase was collected. The bottom aqueous phase was again shaken with 25 ml of MIBK, and the organic phase was obtained and pooled with the previous phase. The pooled organic phase was mixed with 2 ml of 50% HNO_3_, and shaken vigorously for 10 min to separate the bottom acid layer [20]. The marine surface sediment samples were air dried at room temperature and homogenized using an agate mortar and pestle, and sieved using a 63 μm sieve. The particles > 63 μm in size were retained in pre-cleaned and sterilized plastic bottles for trace metal analysis. The trace metals in marine sediment were analyzed as described by Thuy et al. [21] in a slightly changed procedure. One gram of dried sediment was added to 10 ml of ‘aqua regia’ (i.e. HCl:HNO_3_ = 3:1) in Teflon PTFE vessels and treated at 100°C in a hot air oven for 24 h. After incubation, the residue was dissolved with 20 ml of HNO_3_ and filtered in a Millipore filtration unit with 0.45 μm nitrocellulose filter paper. The final volume was made up with HNO_3_ to a volume of 25 ml in a volumetric tube.

The concentration of metals in each water and sediment samples were determined by atomic absorption spectrophotometry (GBC SensAA—AAS, Australia) by a flame method. The blanks and internal standards were run at regular intervals to verify the accuracy of the method. Percent recoveries of metals ranged from 70 to 88% and the relative standard deviation of triplicate measurements was < 5%. Four important parameters [22] were used to evaluate the degree of trace metal contaminations in marine sediments such as enrichment factor (EF), contamination factor (CF), pollution load index (PLI) and geo-accumulation index (*I*_*geo*_).

EF=(Me/Fe)sample/(Me/Fe)background (1)

where, (Me/Fe)_sample_ is the metal to Fe ratio in the sample of interest; (Me/Fe)_background_ is the natural background value of metal to Fe ratio.

$$CF=C_{m}sample/C_{m}background$$ (2)

where, C_m_ Sample is the concentration of a given metal in coastal sediments, and C_m_ Background is the value of the metal equals to the world surface rock average.

$$PLI={\left(CF_{1}XCF_{2}XCF_{3}\ldots ..CFn\right)}_{1/n}$$ (3)

where, n is the number of metals (seven in the present study).

$$I_{geo}=Log2[C_{m}sample/(1.5XC_{m}background)]$$ (4)

where, C_m_ Sample is the measured concentration of elements n in the sediment sample and the C_m_ Background is the geochemical background value. The factor 1.5 is introduced to include possible variation of the background values due to lithogenic effects. The enrichment factor (EF) categories, contamination level of CF and geo-accumulation index classes (seven) are given in S2 Table.

### Statistical analysis

Pearson correlation coefficient and scatter matrix was employed for a better understanding of the relationship between the concentration of multiple variables (trace metals, geochemical, and bacteriological parameters) simultaneously by using the statistical package ORIGIN8.0. The two way analysis of variance (ANOVA) and scatter matrix was employed (ORIGIN8.0) to understand the variation in the variables between different stations, different locations and their interactions. A scatter matrix reveals a correlation pattern between two parameters and also indicates whether those parameters are supporting each other or not. A 45-degree line diagonal plot indicates a strong relation of two variables while the distribution of the plot explains a lower relation between the variables [13]. Principal component analysis (PCA)/ factor analysis (FA) was also performed (SPSS16.0) to trace the source/ pathway of the parameters with respect to the environment.

## Results

### Geochemical analysis

The average mean value of pH was 7.4, 7.9, 7.6 and 7.1 in premonsoon, monsoon, postmonsoon and summer season, respectively (S1 Fig). The EC in water was due to ionization of dissolved inorganic solids and became a measure of calculated TDS. The EC, TDS and salinity ranged from 26000–67800 μS/cm, 12950–48525 mg/L and 20–38 ppt, respectively. In Cuddalore fishing harbor DO and BOD ranged from 3.3–8.3 mg/L and 3.2–7.9 mg/L, and the DO and BOD levels were highly fluctuating during all seasons. Mean level of TA and TH were 162 mg/L and 101.6 mg/L in monsoon; 106 mg/L and 79.1 mg/L in summer; 114 mg/L and 56 mg/L in premonsoon; 92 mg/L and 61 mg/L in postmonsoon.

### Indicators of bacterial populations

**Fig 2:**
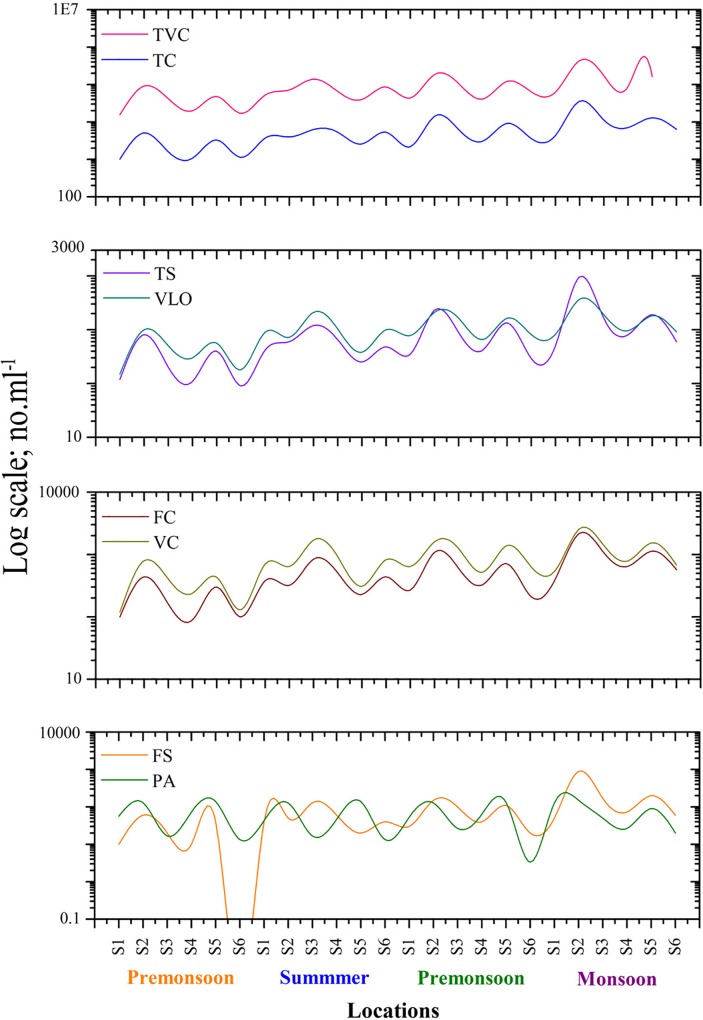
Spatial variation of pollution indicators in water samples.

High levels of pollution indicating bacteria in coastal ecosystems were also indicating a common problem in all investigated areas that often lead to an impairment of beneficial uses and human health. Thus, we represent here the average mean values of all parameters in 144 samples (water and sediment) from the coastlines of both cities for all seasons showing a great variety of distribution patterns. The TVC for water and sediment samples were highest during the monsoon season and least during the postmonsoon. In water samples, the mean TVC ranged from 1.2–12.3 [×104] mL−1 during premonsoon, 4.1–47 [×104] mL−1 during monsoon, 1.3–10.6 [×104] mL−1 during postmonsoon and summer (4.2–16.8 [×104] mL−1) (Fig 2). Variations in the total viable counts (TVC) were large during both seasons and locations. In sediments, the mean TVC were higher 26.0–190.0 [×104] mL−1 during the monsoon and least 13.0–70.0 [×104] mL−1 during the postmonsoon period. Similarly to TVC, the TC, FC, TS and FS ranges were higher during the monsoon than during other seasons. High counts of all bacterial parameters were noticed in Cuddalore fishing harbor (S2) and Pondicherry fishing harbor (S5) due to dense human populations and illegal settlement activities. In sediments, TC was found in the range of 3.1–40.2 [×103] mL−1 during postmonsoon, 17.0–121.0 [×103] mL−1 during monsoon and 1.8–24.7 [×103] mL−1 during premonsoon (S2 Fig). The TS did not show any location-wise trend and the mean densities were low during the postmonsoon. Similar observations were made by Vignesh and co-workers [1] at Tamil Nadu beaches and Kumarasamy et al [14] in the Cauvery estuary, Tamil Nadu.

**Fig 3:**
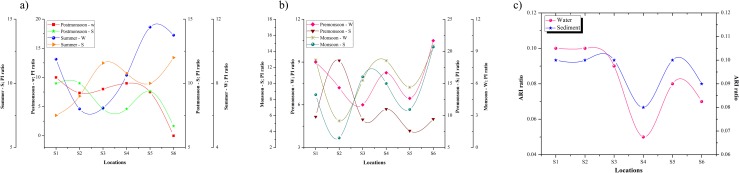
(a and b) Pollution index (PI) ratio; (c) Antibiotic resistant index (ARI) ratio.

In water, FC counts were high at sampling site S2 during all seasons and the values were 1080 mL−1, 2200 mL−1, 440 mL−1 and 320 mL−1 during premonsoon, monsoon, postmonsoon and summer, respectively. Similar patterns were also noted in sediment samples. The VLO counts were higher at the Cuddalore coast than at the Pondicherry coast. In water, the mean values of VLO densities were 1268.33 mL−1, 1685.25 mL−1, 448.33 mL−1 and 1021 mL−1 during premonsoon, monsoon, postmonsoon and summer, respectively. In sediments, the VLO ranges were 2–5 folds higher than in water samples. During monsoon, the counts of VC and PA were generally higher at all sampling sites. Nagvenkar and Ramaiah [4] made similar observations in Mandovi and Zuari estuary at Goa. In water, Cuddalore fishing harbor (S2) showed high VC counts at all seasons i.e., 1700 mL−1, 2600 mL−1, 800 mL−1, 640 mL−1 during premonsoon, monsoon, postmonsoon, and summer, respectively. In sediments, the mean value of VC during monsoon were 1700 mL−1, 7700 mL−1, 3300 mL−1, 2000 mL−1, 3100 mL−1 and 1800 mL−1 at S1, S2, S3, S4, S5 and S6, respectively. In sediments, the counts of *Pseudomonas aeruginosa* (PA) i.e., 30 mL−1, 0 mL−1, 80 mL−1 and 30 mL−1 during postmonsoon, summer, premonsoon and monsoon, respectively, was found at Cuddalore Uppanar estuary (S3). The PI value was above 1 (> 1) indicating that the sampling sites were highly contaminated by human fecal matter rather than animal or other sources (Fig 3A and 3B).

### Antibiotic resistance

A comparison of antibiotic resistant *Pseudomonas* levels of two different coastal cities is provided in Table 1. In this study, antimicrobial resistance patterns showed that the resistance behavior was diverse between the sampling locations and types of samples owing to human fecal matter. In Cuddalore, more than 30% of isolates were resistant to single antibiotics whereas double and 3 antibiotic resistances were recorded in *Pseudomonas* sp. such as 18.3% and 16.6%, respectively. Among a total of 60 *Pseudomonas* isolates, 1.6% were susceptible to all antibiotics in Cuddalore while 21.6% were susceptible to all antibiotics in Pondicherry. The Cuddalore region showed a higher percentage (98.4%) of resistant strains than the Pondicherry region (78.4%) indicating that most of the strains were resistant to one or more of the tested antibiotics. With respect to antibiotic classes, the *Pseudomonas* isolates from the Cuddalore and Pondicherry coast were resistant to 10 types of antibiotics belonging to 7 different groups: β-lactam (23.9% and 17.3%), glycopeptide (56.6% and 46.6%), macrolides (18.3% and 11.6%), aminoglycosides (11.6% and 8.3%), tetracycline (8.3% and 15%), quinolone (0% and 0%) and others (8.3% and 5%). In sea water, antibiotic the resistance index (ARI) was higher at Cuddalore beach (0.10) and Cuddalore fishing harbor (0.10), followed by Cuddalore Uppanar river estuary (0.09), Pondicherry fishing harbor (0.08), Pondicherry Sangarabarani river estuary (0.07), and Pondicherry beach (0.05) (Fig 3C). For both water and sediments, the ARI rate was higher in the Cuddalore region than in the Pondicherry coastal zone, indicating that the accumulation of sewage discharges and fecal matters are higher in the Cuddalore coastal region.

**Table 1:** Two-way analysis of variance (ANOVA) for different parameters.

			Two way- ANOVA				
			Water			Sediment	
		Between	Between		Between	Between	
S.No	Parameters	seasons	locations	Interactions	seasons	locations	Interactions
		*(F value)*	*(F value)*	*(F value)*	*(F value)*	*(F value)*	*(F value)*
1	TVC	325.53*	224.56*	67.77*	211.24*	133.96*	47.37*
2	TC	185.79*	107.69*	41.53*	152.66*	119.39*	43.43*
3	TS	645.81*	575.67*	298.19*	174.08*	148.63*	96.84*
4	FC	211.48*	91.28*	31.59*	197.46*	129.52*	34.50*
5	FS	328.28*	221.61*	138.16**	161.67*	108.10*	81.96*
6	VLO	177.69*	111.66*	36.36*	164.82*	144.51*	36.17*
7	VC	190.45*	118.71*	39.10*	131.24*	123.44*	28.93*
8	PA	0.80#	44.42*	2.38**	3.11**	39.19*	1.79^**#**^
9	Cd	1020.25*	829.45*	99.01*	11.21*	57.29*	5.77*
10	Cr	324.27*	62.69*	13.88*	278.94*	134.01*	19.05*
11	Cu	187.56*	62.69*	13.88*	278.94^**#**^	134.01^**#**^	19.05^**#**^
12	Fe	31.75*	7.79*	2.64**	56.81*	246.89*	24.06*
13	Ni	983.02*	374.84*	133.22*	19.00*	4.96*	0.84^**#**^
14	Pb	10.32*	7.83*	2.49**	352.59*	67.61*	9.66*
15	Zn	44.33*	74.06*	4.29*	206.32*	217.46*	23.37*
16	pH	4.45**	1.80^**#**^	1.40^**#**^	—	—	—
17	EC	30.39*	7.02*	10.86*	—	—	—
18	TDS	9.65*	4.45**	3.65*	—	—	—
19	Salinity	1.05#	5.02*	1.53^**#**^	—	—	—
20	DO	30.17*	10.37*	2.56**	—	—	—
21	BOD	45.01*	8.45*	13.54*	—	—	—
22	TA	99.11*	6.59*	5.00*	—	—	—
2423	TH	1.84#	2.31**	2.64**	—	—	—

* Significant at *p* < 0.001

** Significant at *p* < 0.05

# Not significant

### Metal toxicity

About 120 marine *Pseudomonas* isolates were investigated for their copper resistance from two coastal cities of southern India. The patterns of metal resistance were varying depending on their five different (5, 10, 50, 100 and 250 mM) concentrations. Average concentrations of heavy metals in seawater and sediments were about 50 mM [23]. In the present study, the tube dilution (or) minimal inhibitory concentration (MIC) method confirmed that only few of the 120 isolates could survive at higher (250 mM) concentrations of copper among the remaining isolates. More than 70% of the strains were resistant to minimum levels (5 mM) of metal concentrations whereas a maximum of 4.16% of the strains grew at 250 mM copper concentration. These strains could be used for metal extraction through biosorption. Table 2 provides the percentages of microbial growth (which were producing very distinct effects) and their minimal inhibitory concentration levels against different metal concentrations. The *Pseudomonas* isolates were easily tolerating lower concentrations while higher concentrations strongly affected bacterial growth. A growth rate between 90–100% was observed for 5.8% of the bacterial populations at 5 mM of Cu, whereas no population was growing at a growth rate of 0–40% with 5 mM of Cu. At 10 mM of Cu, 40% of the populations showed a growth rate of 61–70%. In 50 mM, 56.6% of the populations showed growth rates between 51–60%, whereas 33.3% of the populations were observed with a 51–60% growth rate at 100 mM concentrations. At 250 mM Cu, 53.3% of the populations showed 41–50% growth rate. A higher metal resistant *Pseudomonas* sp. was observed at Cuddalore than at Pondicherry.

**Table 2:** Antimicrobial resistant strains and their susceptibilities in the Cuddalore and Pondicherry coastal regions.

Strains	*Pseudomonas* sp.					
	Total strains (*n* = 120)		Cuddalore (*n* = 60)		Pondicherry (*n* = 60)	
	N	%	N	%	N	%
1 Antimicrobial	34	28.3	19	31.6	15	25
2 Antimicrobial	21	17.5	11	18.3	10	16.6
3 Antimicrobial	18	15	10	16.6	8	13.3
4 Antimicrobial	14	11.6	6	10	8	13.3
5 Antimicrobial	8	6.6	5	8.3	3	5
6 Antimicrobial	3	2.5	2	3.3	1	1.6
7 Antimicrobial	4	3.3	2	3.3	2	3.3
8 Antimicrobial	1	0.8	1	1.6	0	0
9 Antimicrobial	2	1.6	2	3.3	0	0
10 Antimicrobial	1	0.8	1	1.6	0	0
Total resistant	106	88.4	59	98.4	47	78.4
Susceptibilities	14	11.6	01	1.6	13	21.6
Antimicrobial agent (10 mcg)	Percentage of *Pseudomonas* sp. resistant to antibiotic					
Ampicillin (AMP)	40	33.3	24	40	16	26.6
Amoxycillin (AMX)	26	21.6	16	26.6	10	16.6
Chloramphenicol (C)	8	6.6	5	8.3	3	5
Ciprofloxacin (CIP)	0	0	0	0	0	0
Erythromycin (E)	18	15	11	18.3	7	11.6
Gentamicin (GEN)	12	10	7	11.6	5	8.3
Methicillin (MET)	44	36.6	24	40	20	33.3
Penicillin—G (P)	68	73.3	51	85	37	61.6
Tetracycline (TE)	14	11.6	5	8.3	9	15
Vancomycin (VA)	48	51.6	34	56.6	28	46.6

N–Numbers; %—Percentage

### Assessment of trace metals

**Fig 4:**
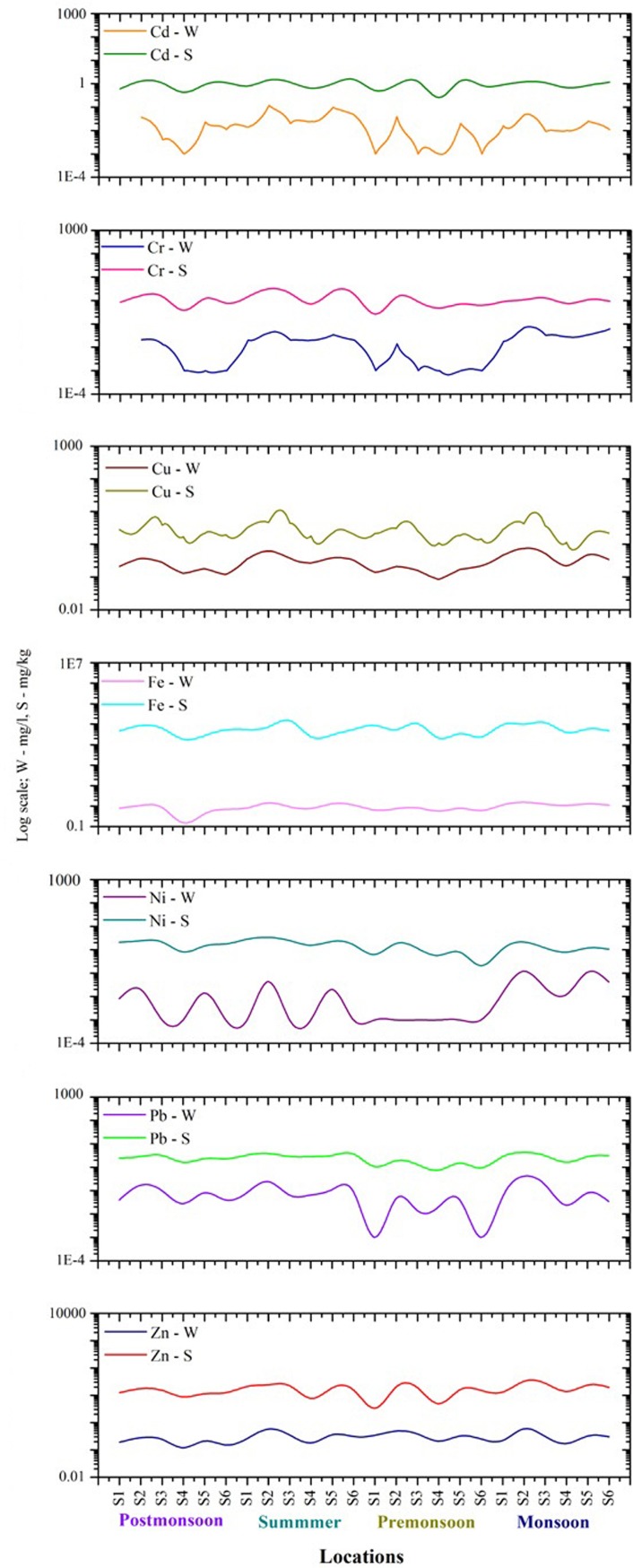
Spatial and temporal variations of trace metals in water and sediment samples.

Concentrations of various trace metals in different water and sediment samples are shown in Fig 4. The ranges of Cd, Cr, Cu, Fe, Ni, Pb and Zn metals in seawater samples were below detectable limits (BDL)– 0.12, BDL– 0.071, 0.085–0.74, 0.15–1.56, BDL– 0.12, BDL– 0.41 and 0.12–0.59 mg L-1, respectively. A minimum concentration (BDL) of Cd was found during premonsoon and postmonsoon while maximum concentrations were observed in summer and monsoon. The mean Cu ranged from 0.085–0.22 mL−1 during premonsoon, 0.22–0.74 mL−1 during monsoon, 0.12–0.37 mL−1 during postmonsoon and 0.27–0.62 mL−1 during summer. Maximum concentrations of trace metals were observed in summer and monsoon followed by postmonsoon and premonsoon. Average concentrations of Cd, Cr, Cu, Fe, Ni, Pb, and Zn were found as 0.024, 0.018, 0.30, 0.91, 0.017, 0.083, and 0.29 mg L-1, respectively.

In marine sediments, levels of trace metals ranged from 0.43–1.45 mg kg-1 for Cd; from 1.1–4.65 mg kg-1 for Cu; from 0.26–1.98 mg kg-1 for Cr; from 1834–14990 mg kg-1 for Fe; from 0.21–3.35 mg kg-1 for Ni; from 0.75–4.28 mg kg-1 for Pb, and from 4.93–32.45 mg kg-1 for Zn. Average concentrations of Cd, Cr, Cu, Fe, Ni, Pb, and Zn were found as 0.95, 1.17, 2.57, 6114.12, 1.55, 2.49, and 16.47 mg kg-1, respectively. Status of sediment pollution was assessed through the Tomilson pollution load index proposed by Tomilson et al. [24]. The world average shale values of Cd (0.3 mg/L), Cr (90 mg/L), Cu (45 mg/L), Fe (47200 mg/L), Ni (68 mg/L), Pb (20 mg/L) and Zn (95 mg/L) were considered as background values [25]. Pollution load index (PLI) values are indicating that the stations were not highly contaminated with trace metals. The variations in CF values are indicating that contamination with Cd in the sediment was highest in comparison with other metals. With respect to the CF class, all values were less than 1 at all stations except Cd. This indicates a low level of sediment contamination in the proposed study area. The CF values were suggesting that these stations were highly polluted with Cd, which was due to its location nearest to the industrial effluents and the aquaculture sewage outfall point of Cuddalore coastal area.

The heavy metal enrichment factor is a measure of untreated effluents and sewage discharges in the aquatic environment. The enrichment factor has been used to examine the anthropogenic contribution of metals in sediments and to differentiate the metal source. This holds for anthropogenic or natural based elements against background levels. At some of the locations, the EF value of Cd was higher than 40, indicating a higher Cd pollution in these regions and, Pb and Zn were crossing the EF value of 2, suggesting that these areas were only moderately polluted (Table 3). However, the enrichment factor values obtained for most heavy metals (Cu, Ni, Fe, Cr & Pb) being less than 1 reveals that these elements were originating from non-anthropogenic heavy metals in the sediments at most of the sampling sites. The *I*_*geo*_ values calculated for Cd (1.69) showed higher values throughout the study period in the industrial zone of Cuddalore and Pondicherry. Interestingly, *I*_*geo*_ values greater than 1 revealed that a significant portion of metals originated from geological sources.

**Table 3:** Percentage of isolated copper resistant strains from the Cuddalore and Pondicherry coastal zone.

**Percentage of growth**	**Copper (Cu)—*Pseudomonas* sp.–(*n* = 120)**									
	5 mM		10 mM		50 mM		100 mM		250 mM	
	N	%	N	%	N	%	N	%	N	%
0–10% of growth	-	-	-	-	-	-	-	-	-	-
10–20% of growth	-	-	-	-	-	-	-	-	-	-
21–30% of growth	-	-	-	-	-	-	-	-	5	4.1
31–40% of growth	-	-	-	-	-	-	10	8.3	25	20.8
41–50% of growth	5	4.1	1	0.8	6	5	52	43.3	64	53.3
51–60% of growth	24	20	44	36.6	68	56.6	40	33.3	20	16.6
61–70% of growth	60	50	48	40	28	23.3	12	10	6	5
71–80% of growth	22	18.3	17	14.1	12	10	-	-	-	-
81–90% of growth	2	1.6	-	-	-	-	-	-	-	-
91–100% of growth	7	5.8	10	8.3	6	5	6	5	-	-
**Locations**	**Minimal inhibitory concentration (MIC) of copper—*Pseudomonas* sp. (*n* = 120)**									
	5 mM		10 mM		50 mM		100 mM		250 mM	
Cuddalore coast (60 N)		107		92		64		26		5
Pondicherry coast (60 N)		96		81		42		13		0

N–Numbers

### Two-way analysis of variance (ANOVA)

The two-way analysis of variance (ANOVA) test examines the influence of different categorial independent variables on one dependent variable. It can not only determine the main effect of contributions of each independent variable but also identifies if there is a significant interaction effect between the independent variables. Differences in the concentration of parameters between the sampling locations were statistically significant (Table 4) which indicated that strong signals of seasonal variations and local variations could be discerned. It was revealed that significant (ANOVA, *P* < 0.001 and *P* < 0.05) variations were observed between different seasons and locations, and the interactions of water and sediments.

**Table 4:** The degree of trace metal contaminations in Cuddalore and Pondicherry coastal sediments.

Seasons	S.S	EF value						*I*_*geo*_ value							CF value							PLI
		Cd	Cr	Cu	Ni	Pb	Zn	Cd	Cr	Cu	Fe	Ni	Pb	Zn	Cd	Cr	Cu	Fe	Ni	Pb	Zn	
**Postmonsoon**	**S1**	14.98	0.05	0.31	0.08	0.70	0.69	1.00	-7.24	-4.62	-2.91	-6.59	-3.42	-3.44	3.00	0.01	0.06	0.20	0.02	0.14	0.14	0.10
	**S2**	19.20	0.06	0.45	0.15	1.02	1.63	1.43	-6.97	-3.99	-2.84	-5.57	-2.81	-2.13	4.03	0.01	0.09	0.21	0.03	0.21	0.34	0.15
	**S3**	14.53	0.06	0.33	0.07	0.63	1.15	1.29	-6.73	-4.19	-2.57	-6.41	-3.24	-2.37	3.67	0.01	0.08	0.25	0.02	0.16	0.29	0.13
	**S4**	26.81	0.10	0.30	0.14	0.98	1.73	0.60	-7.51	-5.88	-4.15	-7.01	-4.18	-3.36	2.27	0.01	0.03	0.08	0.01	0.08	0.15	0.06
	**S5**	22.76	0.10	0.31	0.14	1.14	2.02	0.92	-6.95	-5.27	-3.59	-6.43	-3.40	-2.58	2.83	0.01	0.04	0.12	0.02	0.14	0.25	0.10
	**S6**	39.35	0.11	0.50	0.16	1.63	2.08	1.37	-7.17	-4.94	-3.93	-6.60	-3.23	-2.87	3.87	0.01	0.05	0.10	0.02	0.16	0.20	0.09
**Summer**	**S1**	20.42	01.0	0.64	0.31	1.26	1.34	0.00	0.00	-4.59	-3.94	-5.62	-3.61	-3.52	2.00	0.01	0.06	0.10	0.03	0.12	0.13	0.09
	**S2**	23.60	0.10	0.37	0.19	0.78	1.01	1.53	-6.36	-4.47	-3.03	-5.41	-3.39	-3.02	4.33	0.02	0.07	0.18	0.04	0.14	0.18	0.13
	**S3**	26.54	0.12	0.65	0.24	1.21	1.21	1.22	-6.57	-4.13	-3.51	0.00	-3.23	-3.24	3.50	0.02	0.09	0.13	0.03	0.16	0.16	0.12
	**S4**	36.89	0.11	0.97	0.30	2.08	2.37	0.00	0.00	-5.31	-5.27	0.00	-4.21	-4.03	1.43	0.00	0.04	0.04	0.01	0.08	0.09	0.04
	**S5**	52.92	0.23	0.82	0.36	1.98	2.04	1.05	0.00	-4.97	-4.68	-6.15	-3.69	-3.65	3.10	0.01	0.05	0.06	0.02	0.12	0.12	0.08
	**S6**	33.11	0.08	0.39	0.23	1.02	1.17	1.30	0.00	-5.11	-3.75	0.00	-3.72	-3.52	3.70	0.01	0.04	0.11	0.03	0.11	0.13	0.09
**Premonsoon**	**S1**	22.70	0.14	0.61	0.35	1.41	1.89	0.83	-6.54	-4.40	-3.67	0.00	-3.18	-2.75	2.67	0.02	0.07	0.12	0.04	0.17	0.22	0.11
	**S2**	31.10	0.23	0.66	0.32	1.25	1.64	1.69	-5.40	-3.86	-3.27	-4.93	-2.95	-2.55	4.83	0.04	0.10	0.16	0.05	0.19	0.26	0.18
	**S3**	12.07	0.07	0.31	0.11	0.46	0.73	1.35	-6.09	-3.92	-2.24	0.00	-3.37	-2.69	3.83	0.02	0.10	0.32	0.04	0.15	0.23	0.16
	**S4**	41.03	0.15	0.77	0.44	2.73	1.55	0.51	-7.55	-5.23	-4.85	0.00	-3.40	-4.21	2.13	0.01	0.04	0.05	0.02	0.14	0.08	0.07
	**S5**	53.00	0.43	0.76	0.51	2.50	3.06	1.18	-5.75	-4.95	-4.55	-5.51	-3.22	-2.93	3.40	0.03	0.05	0.06	0.03	0.16	0.20	0.11
	**S6**	42.37	0.17	0.39	0.20	1.55	1.38	1.74	-6.23	-5.04	-3.67	0.00	-3.04	-3.20	5.00	0.02	0.05	0.12	0.02	0.18	0.16	0.12
**Monsoon**	**S1**	9.34	0.02	0.26	0.05	0.29	0.19	0.00	0.00	-4.98	-3.04	0.00	0.00	-5.41	1.70	0.00	0.05	0.18	0.01	0.05	0.04	0.04
	**S2**	26.43	0.14	0.58	0.24	0.82	1.82	1.03	-6.57	-4.47	-3.69	0.00	-3.98	-2.83	3.07	0.02	0.07	0.12	0.03	0.10	0.21	0.11
	**S3**	18.47	0.04	0.25	0.07	0.29	0.88	0.00	0.00	-4.72	-2.75	0.00	-4.53	-2.93	4.13	0.01	0.06	0.22	0.02	0.07	0.20	0.10
	**S4**	19.34	0.12	0.55	0.19	0.84	1.16	0.00	0.00	-5.94	-5.07	0.00	-5.32	-4.82	0.87	0.01	0.02	0.04	0.01	0.04	0.05	0.03
	**S5**	60.84	0.11	0.56	0.16	1.01	2.29	1.57	0.00	-5.19	-4.35	0.00	-4.34	-3.16	4.47	0.01	0.04	0.07	0.01	0.07	0.17	0.07
	**S6**	54.28	0.13	0.63	0.06	0.91	3.05	0.00	0.00	-5.53	-4.86	0.00	0.00	-3.25	2.80	0.01	0.03	0.05	0.00	0.05	0.16	0.04

S.S–Sampling stations

EF value–Enrichment factor value

*I*_*geo*_ value–Geo-accumulation indices value

CF value–Contamination factor value

PLI–Pollution loading index

Descriptive statistics of microbial, physiochemical and trace metal parameters are represented in S3 Table. Results from two-way ANOVA demonstrate that copper in sediment samples had no significant effects in all divisions such as between seasons, between locations and their interactions. In sediment samples, insignificant effects were also observed in PA and Ni at divisions of their interactions (seasons *vs* locations). Similar to this, an insignificant effect was observed with pH (between locations and their interactions) and salinity (between seasons and their interactions) of water samples. Most of the parameters had a significant effect between seasons and between locations and their interactions. No significant (ANOVA, *P* > 0.05) effect was observed in PA, salinity and TH between seasons.

### Correlation and scatter matrix

Correlations between the parameters can provide information on the relationship of the sources and pathways of the factors. Correlations between the variables are listed in Table 5.

**Table 5:** Coefficient correlation on microbial, geochemical and trace metal parameters.

	TVC	TC	TS	FC	FS	VLO	VC	PA	pH	EC	TDS	Sali	DO	BOD	TA	TH	Cd	Cr	Cu	Fe	Ni	Pb	Zn
TVC	1																						
	0																						
TC	0.99*	1																					
	0**	0																					
TS	0.95	0.97	1																				
	3.2E-13	2.6E-15	0																				
FC	0.97	0.97	0.90	1																			
	2.2E-16	2.2E-16	8.5E-10	0																			
FS	0.95	0.96	0.99	0.91	1																		
	4.7E-13	7.5E-15	0	5.2E-10	0																		
VLO	0.95	0.93	0.85	0.96	0.86																		
	4.3E-13	1.5E-11	8.4E-8	3.2E-14	6.9E-8	0																	
VC	0.92	0.90	0.80	0.94	0.81	0.99	1																
	1.2E-10	1.1E-9	1.6E-6	2.8E-12	1.3E-6	0	0																
PA	0.29	0.32	0.36	0.27	0.33	0.24	0.24	1															
	0.16	0.12	0.08	0.19	0.11	0.25	0.25	0															
pH	-0.19	-0.19	-0.14	-0.20	-0.10	-0.22	-0.25	-0.43	1														
	0.34	0.36	0.50	0.34	0.61	0.28	0.23	0.03	0														
EC	0.03	0.04	0.04	0.11	0.06	0.03	0.05	0.16	0.17	1													
	0.88	0.82	0.82	0.62	0.77	0.85	0.79	0.43	0.41	0													
TDS	0.03	0.04	0.04	0.11	0.06	0.03	0.05	0.16	0.17	1	1												
	0.88	0.82	0.82	0.62	0.77	0.85	0.79	0.43	0.41	0	0												
Sali	-0.31	-0.26	-0.27	-0.21	-0.28	-0.24	-0.20	0.21	0.004	0.40	0.40	1											
	0.13	0.20	0.19	0.31	0.17	0.24	0.33	0.31	0.98	0.05	0.05	0											
DO	0.42	0.37	0.34	0.41	0.35	0.45	0.40	0.17	-0.23	-0.22	-0.22	-0.47	1										
	0.03	0.07	0.09	0.04	0.09	0.02	0.04	0.41	0.26	0.29	0.29	0.01	0										
BOD	0.25	0.27	0.22	0.28	0.21	0.28	0.28	0.22	-0.22	0.17	0.17	0.21	-0.03	1									
	0.23	0.19	0.29	0.17	0.30	0.18	0.18	0.28	0.29	0.40	0.40	0.31	0.85	0									
TA	0.67	0.67	0.57	0.73	0.60	0.66	0.66	0.10	0.04	0.12	0.12	-0.11	0.18	0.35	1								
	3.2E-4	2.9E-4	0.003	3.8E-5	0.001	3.5E-4	4.1E-4	0.61	0.82	0.54	0.54	0.57	0.38	0.08	0								
TH	0.18	0.15	0.11	0.15	0.14	0.13	0.15	-0.18	0.12	0.08	0.08	-0.35	-0.05	-0.11	0.17	1							
	0.38	0.47	0.58	0.46	0.49	0.52	0.47	0.39	0.55	0.67	0.67	0.08	0.78	0.59	0.40	0							
Cd	0.41	0.41	0.34	0.39	0.27	0.44	0.46	0.60	-0.60	-0.31	-0.31	-0.02	0.25	0.22	0.11	-0.14	1						
	0.04	0.04	0.09	0.05	0.18	0.02	0.02	0.002	0.001	0.14	0.13	0.89	0.22	0.28	0.60	0.51	0						
Cr	0.78	0.76	0.69	0.77	0.69	0.72	0.70	0.23	-0.10	-0.12	-0.12	-0.25	0.40	0.24	0.65	0.21	0.49	1					
	6.8E-6	1.2E-5	1.4E-4	7.1E-6	1.7E-4	5.3E-5	1.2E-4	0.27	0.62	0.54	0.54	0.22	0.05	0.23	4.9E-4	0.30	0.01	0					
Cu	0.80	0.78	0.71	0.80	0.70	0.79	0.76	0.34	-0.28	-0.12	-0.12	-0.16	0.55	0.37	0.63	0.06	0.57	0.83	1				
	2.6E-6	6.4E-6	8.1E-5	2.1E-6	1.1E-4	3.1E-6	1.2E-5	0.09	0.17	0.56	0.56	0.44	0.004	0.07	6.9E-4	0.74	0.003	4.3E-7	0				
Fe	0.68	0.66	0.57	0.68	0.56	0.67	0.65	0.25	-0.19	-0.31	-0.31	-0.33	0.42	0.12	0.56	0.17	0.60	0.82	0.86	1			
	1.9E-4	3.8E-4	0.003	2.1E-4	0.003	2.8E-4	4.8E-4	0.23	0.35	0.12	0.12	0.10	0.03	0.55	0.004	0.42	0.001	8.1E-7	5.9E-8	0			
Ni	0.80	0.83	0.78	0.82	0.79	0.71	0.69	0.35	-0.10	0.12	0.12	-0.20	0.25	0.25	0.68	0.38	0.33	0.74	0.73	0.66	1		
	1.7E-6	4.8E-7	4.8E-6	5.4E-7	3.6E-6	8.9E-5	1.4E-4	0.09	0.61	0.57	0.57	0.34	0.22	0.23	2.1E-4	0.06	0.11	2.6E-5	4.5E-5	4.1E-4	0		
Pb	0.88	0.87	0.87	0.83	0.84	0.83	0.79	0.41	-0.35	-0.06	-0.06	-0.15	0.40	0.33	0.48	0.15	0.56	0.70	0.84	0.67	0.68	1	
	6.4E-9	1.7E-8	3.0E-8	3.0E-7	1.5E-7	4.0E-7	3.1E-6	0.04	0.08	0.75	0.75	0.46	0.04	0.11	0.01	0.47	0.003	1.1E-4	2.5E-7	2.6E-4	2.4E-4	0	
Zn	0.63	0.61	0.61	0.55	0.57	0.57	0.53	0.42	-0.42	-0.18	-0.18	-0.44	0.57	-0.06	0.13	0.03	0.64	0.55	0.63	0.61	0.50	0.64	1
	8.2E-4	0.001	0.001	0.004	0.003	0.003	0.007	0.03	0.04	0.39	0.39	0.02	0.003	0.75	0.52	0.86	7.0E-4	0.004	9.6E-4	0.001	0.01	6.7E-4	0

Sali–Salinity

*—Correlation value

**—Significance value

High positive correlations were observed between bacterial parameters except PA with other bacterial parameters. This indicates that bacterial groups were strongly associated with each other ('*p*'*value*–<0.0001) due to their same pollution origin/ sources. Interestingly, the geochemical parameters were not associated with bacterial parameters except TA-bacterial parameters. At the same time, heavy metal parameters were positively associated with each other and their parameters were highly significant ('*p*'*value*–<0.05), except Cd-Cr, Ni & Zn-Ni. A poor correlation between TH, TA and heavy metals may have resulted from a tremendous deviation in the content of those parameters which proved that they were affected by different sources. A great relocation difference was also observed with physiochemical parameters. Commonly, less positive and negative correlations were observed between salinity and all other parameters which confirmed that they were not mutually associated with each other. A similar observation was also observed between pH and all other parameters. However, bacterial parameters were mutually associated with heavy metal parameters confirming that the minimum level of trace metals were acting as micronutrients and were supporting microbial growth with high significances ('*p*'*value*–<0.05).

**Fig 5:**
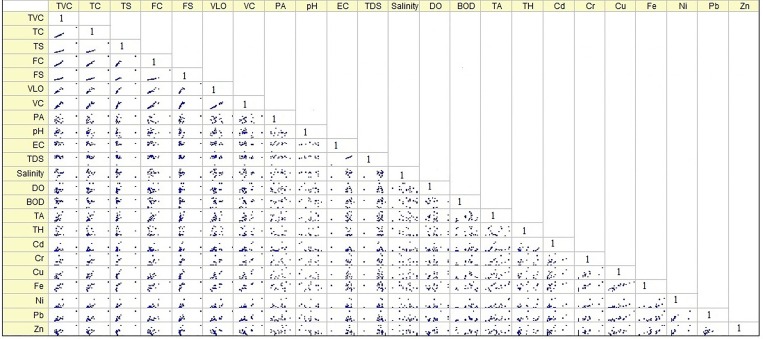
Scatter matrix plot for interrelationship of microbial, physiochemical and trace.

The scatter matrix (Fig 5) explained that the distribution pattern of parameter levels and the linear line distribution indicated that parameters were mutually associated with each other. This pattern was also used to estimate the co-variance matrix for example of the normal distribution of multi-variance. The pair-wise plots are explaining the univariate distribution of the variables and also indicate the deviated values which are not plotted on the 45-degree liner line. The scatter matrix showed a strong association between the bacterial parameters except PA and a similar pattern is also followed between the Cu and Fe; EC and TDS. While dispersed values were observed between the PA, pH, DO, BOD, TA and TH = all parameters (except TVC, TC, TS, FC, FS, VLO and VC). But, a moderate level in the 45° diagonal plot was observed between the remaining parameters especially between trace metals.

### Factor analysis

To evaluate the possible relationships among variables, we performed statistical analysis such as principle component analysis (PCA)/ factor analysis (FA) with varimax normalization (PCA-V) to explore the sources and pathways of the variables. In this study, a factor with an eigen *value* > 1 was considered for subsequent discussion (Table 6). The factor I explained 48.86% of the total variance and was best represented by bacterial parameters (except PA), heavy metals (expect Cd and Zn) and TA. This factor represents a pollution source from sewage and fecal contaminations throughout the coastal regions and it could be considered as a ‘microbe–metal’ interaction factor. An overwhelming 13.23% of the total variance was contributed by factor II, showing higher loading for geochemical parameters such as EC (0.929), TDS (0.929), and salinity (0.625) (except pH, DO, BOD, TA and TH), which is considered as an ‘anthropogenic’ factor. Factor 3 explained 11.74% of the total variation and has a positive loading on PA and Cd, a moderate positive loading on BOD, whereas a high negative loading on pH and TH is considered as a ‘*Pseudomonas*–Cadmium’ factor.

**Table 6:** Mean and principle component analysis (PCA) for microbial, geochemical and trace metal parameters.

Variable	Mean	Standard deviation	Communalities	Factor I	Factor II	Factor III
TVC	9.4950E4	87878.73	0.968	**0.979**	-0.056	0.082
TC	6.6754E3	7247.52	0.956	**0.973**	-0.009	0.099
TS	1.0800E3	1904.73	0.860	**0.923**	-0.004	0.088
FC	5.4750E2	467.92	0.962	**0.977**	0.035	0.083
FS	98.750	174.66	0.865	**0.929**	0.011	0.030
VLO	1.1058E3	801.13	0.892	**0.936**	-0.037	0.120
VC	8.6708E2	591.99	0.844	**0.909**	-0.010	0.134
PA	74.166	51.57	0.640	0.270	0.195	**0.728**
pH	7.4250	0.52	0.584	-0.115	0.178	*-0*.*734*
EC	5.1653E4	12736.81	0.828	0.097	**0.899**	-0.105
TDS	3.2541E4	8024.49	0.828	0.097	**0.899**	-0.105
Salinity	28.8750	3.50	0.663	-0.299	**0.666**	0.360
DO	5.2104	1.32	0.404	0.415	-0.457	0.153
BOD	6.5954	1.67	0.337	0.282	0.370	0.348
TA	1.1740E2	30.83	0.617	**0.755**	0.156	-0.149
TH	58.5958	35.62	0.352	0.279	-0.063	*-0*.*520*
Cd	0.0240	0.03013	0.819	0.362	-0.311	**0.769**
Cr	0.0186	0.01965	0.734	**0.827**	-0.206	0.091
Cu	0.3081	0.16748	0.826	**0.834**	-0.185	0.310
Fe	0.9146	0.33388	0.735	**0.727**	-0.416	0.183
Ni	0.0178	0.03260	0.766	**0.873**	0.058	0.002
Pb	0.0837	0.09081	0.842	**0.853**	-0.100	0.322
Zn	0.2971	0.12203	0.661	0.583	-0.419	0.383
Eigen value	—	—	—	11.239	3.044	2.701
% of variance	—	—	—	48.865	13.237	11.745
Cumulative %	—	—	—	48.865	62.101	73.846

PCA loadings > 0.60 (significantly positive) are shown in bold

Significantly negative (> - 0.60) values are shown in italics

The underlined values are not loaded in any factor (which is not loading as either positive or negative values in all the factors)

## Discussion

A rapid increase of anthropogenic activities in coastal areas has caused a significant increase of pollutants and direct impacts on coastal ecosystems, particularly in vulnerable regions. Accumulation of pollutants in coastal ecosystems degrade environmental quality and deplete environmental filtering and self-purification capacity [26]. Microbial indicators have been used worldwide to indicate whether human/ animal wastes have contaminated a water body. The present study was conducted to evaluate the microbial (pollution indicator loads and antibiotic resistant and trace metal resistant strain loads) and metal pollution in populated coastal cities (Cuddalore and Pondicherry). This study was also focusing on the relationship between pollutants, both season- and location-wise. Moreover, the two-way ANOVA, correlation and PCA were helpful to determine whether the variables were significantly different between season and location, as well as their interactions. These statistical tools were shown to be useful for an effective understanding and interpretation of the different variables belonging to three different parameters from both samples.

Total coliforms comprise a large group of organisms of which a major proportion detected in water samples might be of fecal origin, indicating that total coliforms might be a reliable indicator for fecal contamination [27]. The World Health Organization [28] recommended that fecal coliform counts must not exceed 1000/ 100 ml for intermediate contact and 130/ 100 ml for full recreational contact. In the present study from Indian waters, the coliform and *Streptococci* were lowest during post monsoon and highest during monsoon. A similar pattern was also reported in Indian coastal zones such as Tamil Nadu beaches [1], Cauvery river estuary [14], Tamirabarani river estuary [13], Mandovi and Zuari estuary [4], Gujarat waters [29], the Visakhapatnam coast [3], and Mumbai waters [30].

The fishing harbors (S2 and S5) of both cities received more pollution due to dense human populations and the confluence of a variety of discharges and fecal contaminations. Counts of coliforms observed from Cuddalore and Pondicherry areas were at least 2–5 orders of magnitude lower than in Mumbai harbor waters [30], and 1–2 fold lower at Tamil Nadu beaches [1]. The counts of pathogens from Uppanar (S3) and Sangarabarani (S6) estuaries were at least 1–3 orders lower than in the Seine estuary [31]. However, the counts of pathogens were higher in the Cauvery estuary [14,15] than in the S3 and S6 regions. When compared to the Mandovi and Zuari estuary [4], bacterial counts were 1–3 orders lower in the present report. Compared to the Uppanar and Sangarabarani estuary, total coliform and pathogen counts were 5–7 orders higher in the Coovum estuary in Chennai [2].

However, sediments get resuspended due to various biogenic and physical disturbances and sediments may contain 100 to 1000 fold higher bacterial counts than the overlying water layers [5]. The higher bacterial counts in sediments are due to autochthonous action [4], bacterial aggregations by dynamic flocculation [1], rich organic content [13], waves, wind, temperature, and tidal activities [2]. Co-sedimentation, i.e., the joint deposition of sediment particles and bacteria is a common way of bacterial settlement in moving waters. In addition to microorganisms, urine and feces containing organic matter as well as eutrophying substances in the form of phosphorus and nitrogen compounds [32]. Similar results were observed in rivers and coastal waters [1,13]. Thus, higher accumulation of physiochemical and trace metal parameters could not enhance microbial growth.

The fecal matters from humans and animals can easily settle in sediment and this protects microorganisms and also enhances their survival [33]. Other nonpoint sources of contamination (malfunctioning sewage, septic systems, storm water drainage, and urban runoff) could also settle in the sediment, leading to bioaugmentation. By limits set by the United States Environmental Protection Agency (USEPA), recreational waters with concentrations exceeding the maximum contaminant limits of 33 or 200 cfu/100 ml for FC, respectively, presents a health risk [34]. Fecal coliform and *Streptococci* ratios of the study sites showed a pollution index (PI) ratio of more than one [35] which indicated that the sampling sites were possibly contaminated by human excretions. The average PI ratio of the entire study area further indicated specific variations among the locations with all-time occurrences of indicator organisms and it proved that the sampling locations were contaminated by human feces during all seasons.

Marine isolates were highly resistant to antibiotics when compared to clinical isolates [36]. The improper and overuse of antibiotics has promoted the emergence of resistant strains that provided a selective pressure on bacterial strains, and caused an imbalance between susceptible and resistant bacteria [2]. In the recent past, unnecessary usage of antibiotics by humans and animals for various purposes led to changes in microbial genetic ecology through mutation, selection, and the flux of genetic information (horizontal gene transfer) among microbes [37]. The presence of multi-drug resistant strains is alarming because infection with such strains leads to a higher fatality rate than with antibiotic-sensitive strains [38]. Highest antibiotic resistance was observed in marine bacterial isolates from different water and sediment samples of both regions investigated here. More than 85% were resistant to one or more of the tested antibiotics, whereas nearly 10% of the strains were susceptible to the antibiotics tested.

The Cuddalore region got higher resistant strains (98%) than the Pondicherry region (78%) due to municipal discharges, fishing harbor waste and high population density. In both regions, a low frequency (9%) of antibiotic resistance to 6–10 antibiotics was observed in marine *Pseudomonas* isolates. The highest frequency (33%) of antibiotic resistance to 1–3 antibiotics was observed in Cuddalore strains, whereas 27% of antibiotic resistance to 1–3 antibiotics was observed in Pondicherry strains. A minimal percentage of these organisms showed resistance to 4–5 antibiotics. The antibiotic resistance index (ARI) indicated that the Cuddalore region was higher exposed than the Pondicherry region due to an accumulation of municipal sewage, as well as open defecation from human settlements and animal husbandries. In the present study, a higher ARI ratio corresponded to high-risk antibiotic-exposed sites. This indicates that a system understanding is needed in order to prevent the spread of antibiotic resistant bacteria in the marine environment.

Aquatic bacteria can develop resistance against organic matter, inorganic matter, metals and antibiotics by adapting themselves to survive in extreme environments. Microorganisms exposed to selection pressures by toxic compounds develop resistance rapidly [39] and the resistance may vary depending on metal concentrations. In the present study, plate diffusion and tube dilution (MIC) methods confirmed higher sensitivity of *Pseudomonas* sp. growth against high copper concentrations (250 mM). Heavy metals generally inhibit microbial growth by blocking essential functional groups, displacing essential metal ions, or modifying the active conformation of biological molecules [23]. The *Pseudomonas* isolates from fishing harbor areas were much more resistant than in other areas which received different waste materials from shipping activities, domestic waste from slums, paintings, cargo/ ballast water discharges. The mechanism of metal resistance may be affected in two different ways: protein-metal associations–biomagnification or blockage of cell walls and the systems of membrane transportation [40]. However, in the Cuddalore and Pondicherry regions, the percentage of Cu sensitive strains at 5, 10, 50, 100 and 250 mM concentrations were 11.9%, 23.4%, 46.7%, 78.4% and 95.84% and, 20.0%, 32.5%, 65.0%, 89.2% and 0%, respectively. Metal resistant bacteria detoxify metals with the aid of chaperones such as metalloproteins which reduce metal toxicity in water [23]. Above 60% of the strains were resistant to minimum levels of metal concentrations whereas a maximum of 4.16% of the strains grew at 250mM of copper. Heavy metal resistant strains were high in fishing harbors followed by estuaries and beaches in both cities due to the accumulation of several trace metal wastes in coastal sediments.

Increased accumulation of anthropogenic toxic metals in marine environments (fishing harbour, estuary and beach) of both cities from industrial and municipal sources stimulated their extreme persistence and high toxicity [41,42]. In the present study, variations of metal concentrations were compared between both water and sediment samples and were also compared season- and location-wise. The metal concentrations were low in most of the places when compared to the permissible limits of international standards [43,44,45] with some exceptions. The metal concentrations at all the sites from both cities did not show any particular trend and it was indicated that the sources might be diffuse. The abundance of metals in the study regions were in the order Fe > Zn > Cu > Cd > Pb > Ni > Cr. One can observe that the areas influenced by fishing have higher concentrations of toxic metals than other areas throughout the year. The metal concentrations in fishing harbor areas were probably related to nonpoint source influxes from various discharges such as dredging, cargo handling, dumping of ship wastes, spilling of cargo, chemicals, metal ores, ballast water and paint waste directly into the sea [2]. Sewage and urban waste water from town and several settlements, constitute other discharges that are negatively affecting water quality [46]. Similar to the fishing harbors, the Cuddalore estuary (S3) recorded higher values of all metals. The higher concentrations at this site might be due to contributions from agricultural farms and fishing communities dotted along the banks of this river that contained agrochemicals, industrial wastes, and municipal sewage.

Cadmium is extremely toxic and high levels of Cd in water could cause adverse health effects to consumers such as renal diseases and cancer [47]. The WHO has established a human health based guideline of 0.003 mg/L for direct usage [48]. These guidelines were exceeded at some of the sampling sites. The WHO recommended value for Zn in water for domestic supplies is 3 mg/L [48] and the Zn level was not crossing the limits in most places of our study. Copper concentrations were exceeding permissible limits (0.02 mg/L) at certain sites due to surface runoff, fishing boat paints and contributions of river and pipeline discharges to the coastal system. It was shown that concentrations of Ni and Pb were high in some of the places at all seasons and exceeded maximum permissible limits (0.01 and 0.1 mg/L, respectively) at a few sampling sites.

In the factor analysis, factor 1 (48.86%) was influenced by bacterial parameters (except PA), heavy metals (expect Cd and Zn) and TA. Similar observations were recorded along the Chennai coast (Santhiya et al., 2012) and our earlier studies also proved that microbial and physiochemical parameters were not influenced by the same factor [1,13]. Factor 2 (13.23%) was contributed by geochemical parameters. The strong loading of these parameters could be explained by anthropogenic activities through sewage/ industrial/ agricultural/ shipping/ fish-catching activities, clearing of lands, runoff, and erosive processes taking place near the study area [49]. Factor 3 (11.74%) explains the PA and Cd loading with negative pH indicating that PA and Cd levels were fluctuating by precipitation of inorganic substances in this transient coastal aquatic system [50]. These parameters are indicators of organic pollution and the BOD implies that microorganisms utilized DO as biodegradable organic matter [51]. This is also reflecting the control of different sources like anthropogenic discharges with simple hardness association [4]. The DO and BOD did not show either high positive nor high negative values in all the factors. Elevated concentrations of organic substances strengthened anoxia or hypoxia by raising the BOD, the oxygen consumed during microbial decomposition and other organic reduced components [52].

High concentrations of trace metals caused severe health hazards to marine micro- and macrofauna. It may affect their morphology, physiology, biochemistry, enzyme activity, behavior, reproductive system, and causing skin diseases among others. The variation of the enrichment factor (EF) for Cd was significant and the EF values ranged from 9.34–60.84; most of the remaining metals have EF values below 1. This indicates that the sampling stations were more contaminated by cadmium than by other metals. The same pattern was also followed in the geo-accumulation index (*I*_*geo*_), the cadmium level (range = 0.00–1.74) indicated that the sites were moderately polluted. Most of the contamination factor (CF) values for Cd were above 1 (except S4 during the monsoon season) (range = 0.87–4.83), demarcating that the sampling sites were considerably contaminated. The pollution load index (PLI) (range = 0.03–0.18) clearly explained that the sampling sites were below the baseline pollution category (Tomlinson et al., 1980), providing subsequent bioaccumulation in marine biota.

## Conclusion

The studied levels of different pollutants (fecal indicators, antibiotic/ heavy metal resistant strains, physiochemical factors, and trace metals) from water and sediment samples of southern India endowed with major sources of pollution and the levels of bacteria and trace metals were crossing permissible limits of WHO and TNPSC. Different statistical approaches confirmed multiple heterogeneous but also independent sources and pathways of geochemical, microbial, and trace metal parameters. This antibiotic resistance and trace metal study revealed that most of the marine *Pseudomonas* isolates were resistant to at least one antibiotic. The present study in particular calls for the monitoring and subsequent management of several anthropogenic problems that will be necessary to solve in the frame of sustainable coastal management.

## Supporting Information

S1 Fig Seasonal variation of geochemical parameters at the Cuddalore and Pondicherry coast. (TIF)

S2 Fig Spatial variation of pollution indicators in sediment samples. (TIF)

S1 Table Details of specific culture media used for quantitative bacterial analysis. (DOC)

S2 Table Different classifications of contamination levels of trace metals in sediments. (DOC)

S3 Table Descriptive statistics of microbial, geochemical and trace metal parameters. (DOC)
