# Supplementary figures and images for: Biomonitoring along the Tropical Southern Indian Coast with Multiple Biomarkers
Source: PLoS One. 2016 Dec 12;11(12):e0154105. doi: 10.1371/journal.pone.0154105 (PMC5152820; doi:10.1371/journal.pone.0154105)

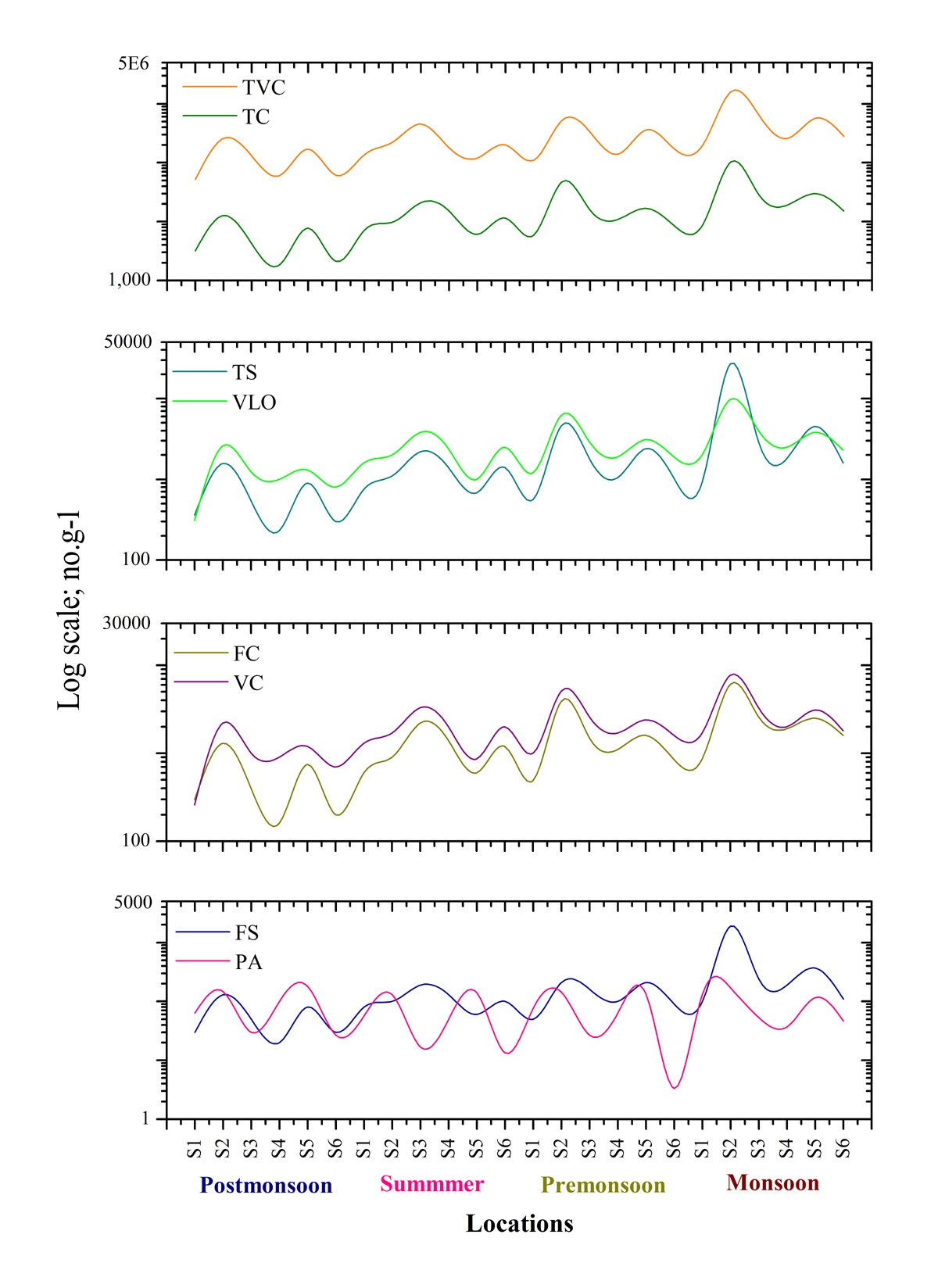

Supplement: S1 Fig — (TIF) [file pone.0154105.s001.tif]

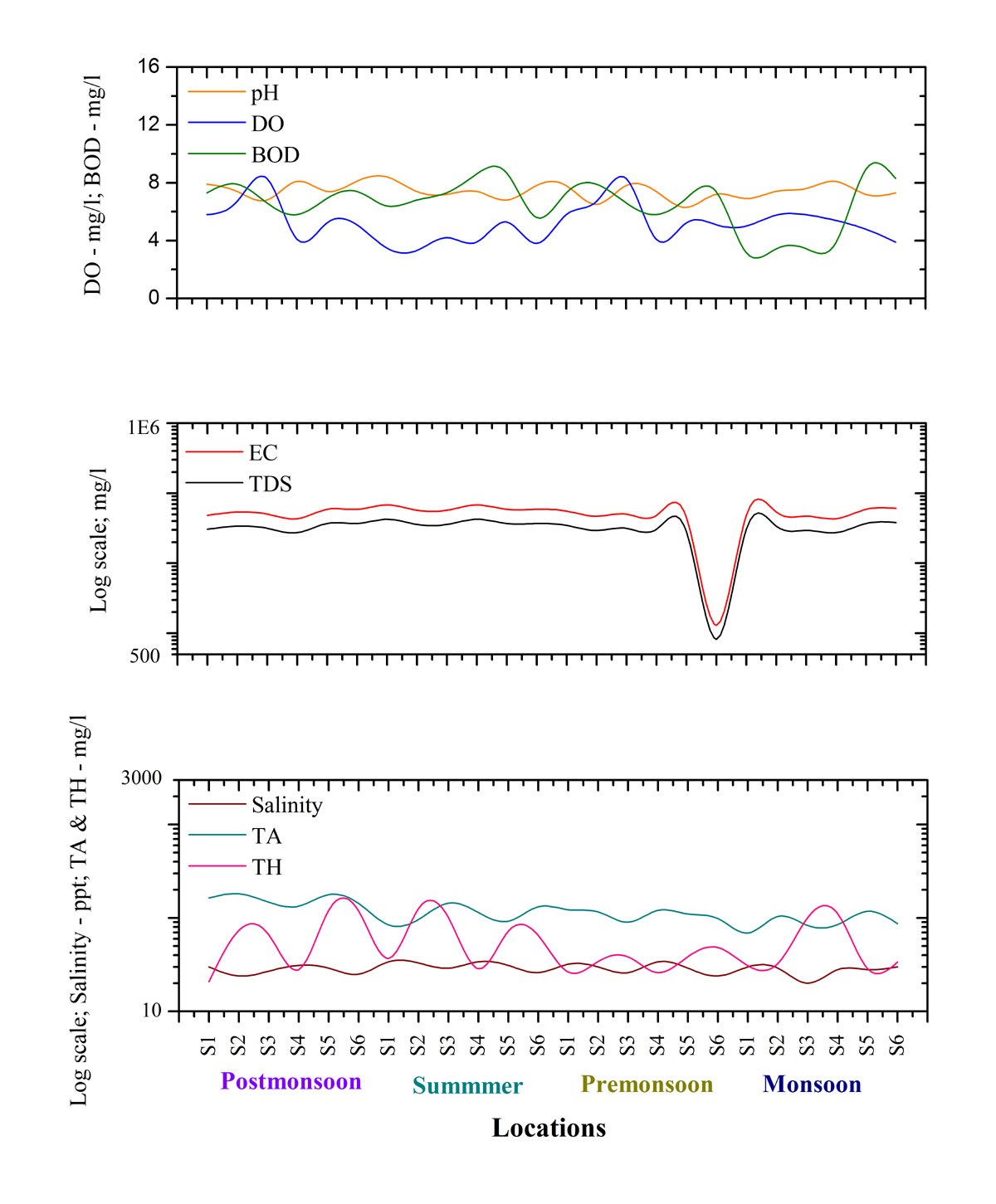

Supplement: S2 Fig — (TIF) [file pone.0154105.s002.tif]
