# Supplementary material for: Biomonitoring along the Tropical Southern Indian Coast with Multiple Biomarkers
Source: PLoS One. 2016 Dec 12;11(12):e0154105. doi: 10.1371/journal.pone.0154105 (PMC5152820; doi:10.1371/journal.pone.0154105)
Supplement: S1 Table — (DOC) [file pone.0154105.s003.doc]

Supplementary table 1. Details of specific culture media used for quantitative bacterial analysis

**S.No Bacterial Indicators Culture medium Positive Colonies Incubation**

1. Total Viable Count **(TVC)** Nutrient Agar All colonies counted 37 °C ± 1°C for 24 to 48 h

2. Total Coliforms **(TC)** MacConkey Agar All colonies counted 37 °C ± 1°C for 24 to 48 h

3. Total *Streptococci* **(TS)** M Enterococcus Agar All colonies counted 37 °C ± 1°C for 24 to 48 h

4. Vibrios Like Organisms **(VLO)** TCBS Agar All colonies counted 37 °C ± 1°C for 24 to 48 h

5. Fecal Coliforms **(FC)** M FC Agar Blue colonies counted 44.5 °C ± 1°C for 24 to 48 h

6. Fecal *Streptococci* **(FS)** KF Streptococcus Agar Red colonies counted 37 °C ± 1°C for 24 to 48 h

7. *Vibrio cholerae* **(VC)** TCBS Agar Yellow colonies counted 37 °C ± 1°C for 24 to 48 h

8. *Pseudomonas aeruginosa* **(PA)** Cetrimide Agar Green colonies counted 37 °C ± 1°C for 24 to 48 h
