# Supplementary material for: Biomonitoring along the Tropical Southern Indian Coast with Multiple Biomarkers
Source: PLoS One. 2016 Dec 12;11(12):e0154105. doi: 10.1371/journal.pone.0154105 (PMC5152820; doi:10.1371/journal.pone.0154105)
Supplement: S2 Table — (DOC) [file pone.0154105.s004.doc]

Supplementary table 2. Different classification for finding the contamination level of trace metals in sediments

| **S.No** | **Categories** |  | **Prescribed levels** |  | **Class / Division** |  | **Quality level** |
| --- | --- | --- | --- | --- | --- | --- | --- |
| 1. | **Geo-accumulation index (I_geo_)** |  | ≤ 0 |  | 0 |  | Unpolluted |
|  |  |  | 0 – 1 |  | 1 |  | From unpolluted to moderately polluted |
|  |  |  | 1 – 2 |  | 2 |  | Moderately polluted |
|  |  |  | 2 – 3 |  | 3 |  | From moderate to strongly polluted |
|  |  |  | 3 – 4 |  | 4 |  | Strongly polluted |
|  |  |  | 4 – 5 |  | 5 |  | From strongly to extremely polluted |
|  |  |  | > 6 |  | 6 |  | Extremely polluted |
|  |  |  |  |  |  |  |  |
| 2. | **Enrichment factor (EF)** |  | EF < 2 |  | -- |  | Deficiency to minimal enrichment |
|  |  |  | 2 ≤ EF < 5 |  | -- |  | Moderate enrichment |
|  |  |  | 5 ≤ EF < 20 |  | -- |  | Significant enrichment |
|  |  |  | 20 ≤ EF < 40 |  | -- |  | Very high enrichment |
|  |  |  | EF ≥ 40 |  | -- |  | Extremely high enrichment |
|  |  |  |  |  |  |  |  |
| 3. | **Contamination factor (CF)** |  | CF < 1 |  | -- |  | Low contamination |
|  |  |  | 1 ≤ CF < 3 |  | -- |  | Moderate contamination |
|  |  |  | 3 ≤ CF < 6 |  | -- |  | Considerable contamination |
|  |  |  | CF > 6 |  | -- |  | Very high contamination |
|  |  |  |  |  |  |  |  |
| 4. | **Pollution load index (PLI)** |  | < 1 |  | -- |  | Baseline pollution |
|  |  |  | > 1 |  | -- |  | Polluted |
