# Supplementary material for: Biomonitoring along the Tropical Southern Indian Coast with Multiple Biomarkers
Source: PLoS One. 2016 Dec 12;11(12):e0154105. doi: 10.1371/journal.pone.0154105 (PMC5152820; doi:10.1371/journal.pone.0154105)
Supplement: S3 Table — (DOC) [file pone.0154105.s005.doc]

Supplementary table 3. Descriptive statistics of microbial, geochemical and trace metal parameters.

| **Parameters** | **N total** | **Mean** | **SD** | **SE** | **L - 95%** | **U - 95%** | **Variance** | **Sum** | **Skewness** | **Kurtosis** | **CV** | **MAD** | **Mode** | **Mini** | **Med** | **Max** |
| --- | --- | --- | --- | --- | --- | --- | --- | --- | --- | --- | --- | --- | --- | --- | --- | --- |
| pH - w | 72 | 7.52 | 0.72 | 0.08 | 7.36 | 7.69 | 0.51 | 541.7 | 0.03 | 1.85 | 0.10 | 0.54 | 7.2 | 5.2 | 7.45 | 9.8 |
| EC - w | 72 | 51044.60 | 13535.64 | 1595.19 | 47863.88 | 54225.32 | 183213000.00 | 3.68E+06 | -2.33 | 6.34 | 0.27 | 8222.78 | 51960 | 1299 | 51960 | 69280 |
| TDS - w | 72 | 32978.52 | 10958.44 | 1291.46 | 30403.41 | 35553.63 | 120087000.00 | 2.37E+06 | -0.48 | 5.37 | 0.33 | 6204.91 | 32734 | 818.3 | 33825 | 69280 |
| Salinity - w | 72 | 28.81 | 3.48 | 0.41 | 27.99 | 29.62 | 12.13 | 2074 | -0.13 | 0.54 | 0.12 | 2.66 | 30 | 20 | 29 | 38 |
| DO - w | 72 | 5.33 | 1.42 | 0.17 | 5.00 | 5.66 | 2.01 | 383.8 | 0.32 | -0.25 | 0.27 | 1.12 | 5.8 | 2.8 | 5.2 | 8.9 |
| BOD - w | 72 | 6.42 | 1.77 | 0.21 | 6.00 | 6.83 | 3.13 | 462.19 | -0.93 | 0.13 | 0.28 | 1.35 | 7.2 | 2.1 | 6.8 | 9.4 |
| TA - w | 72 | 119.23 | 32.62 | 3.84 | 111.56 | 126.89 | 1064.38 | 8584.2 | 0.53 | -0.60 | 0.27 | 26.79 | 76.5 | 67 | 114.2 | 190.6 |
| TH - w | 72 | 74.59 | 77.72 | 9.16 | 56.32 | 92.85 | 6040.31 | 5370.2 | 5.38 | 36.87 | 1.04 | 42.04 | 28.6 | 20.7 | 54.65 | 628.8 |
| TVC - w | 72 | 94950.00 | 87296.85 | 10288.03 | 74436.25 | 115463.75 | 7620740000.00 | 6.84E+06 | 2.69 | 8.68 | 0.92 | 56645.83 | 72000 | 13500 | 72000 | 470000 |
| TC - w | 72 | 6537.92 | 7217.16 | 850.55 | 4841.97 | 8233.87 | 52087400.00 | 470730 | 3.06 | 10.78 | 1.10 | 4436.22 | 2200 | 860 | 4450 | 40000 |
| TS - w | 72 | 1080.00 | 1882.56 | 221.86 | 637.62 | 1522.38 | 3544020.00 | 77760 | 4.04 | 16.57 | 1.74 | 960.83 | 90 | 70 | 555 | 10200 |
| FC - w | 72 | 547.50 | 468.30 | 55.19 | 437.45 | 657.55 | 219309.15 | 39420 | 2.09 | 5.48 | 0.86 | 328.54 | 650 | 80 | 420 | 2500 |
| FS - w | 72 | 98.47 | 173.21 | 20.41 | 57.77 | 139.17 | 30001.86 | 7090 | 4.07 | 16.97 | 1.76 | 88.31 | 40 | 0 | 50 | 970 |
| VLO - w | 72 | 1105.83 | 801.27 | 94.43 | 917.55 | 1294.12 | 642027.46 | 79620 | 1.60 | 3.35 | 0.72 | 597.66 | 900 | 130 | 885 | 4200 |
| VC - w | 72 | 867.36 | 591.51 | 69.71 | 728.36 | 1006.36 | 349884.49 | 62450 | 1.20 | 1.54 | 0.68 | 459.41 | 120 | 120 | 700 | 2900 |
| PA - w | 72 | 70.28 | 54.25 | 6.39 | 57.53 | 83.03 | 2943.58 | 5060 | 0.26 | -1.20 | 0.77 | 47.26 | 0 | 0 | 60 | 190 |
| Cd - w | 72 | 0.02 | 0.03 | 0.00 | 0.02 | 0.03 | 0.00 | 1.728 | 2.01 | 3.89 | 1.24 | 0.02 | 0 | 0 | 0.015 | 0.13 |
| Cr - w | 72 | 0.02 | 0.02 | 0.00 | 0.01 | 0.02 | 0.00 | 1.338 | 1.14 | 1.06 | 1.06 | 0.02 | 0 | 0 | 0.018 | 0.08 |
| Cu - w | 72 | 0.31 | 0.17 | 0.02 | 0.27 | 0.35 | 0.03 | 22.185 | 0.99 | 0.67 | 0.55 | 0.14 | 0.13 | 0.08 | 0.275 | 0.85 |
| Fe - w | 72 | 0.90 | 0.35 | 0.04 | 0.82 | 0.98 | 0.12 | 64.85 | 0.12 | -0.02 | 0.39 | 0.28 | 0.71 | 0.13 | 0.85 | 1.8 |
| Ni - w | 72 | 0.02 | 0.03 | 0.00 | 0.01 | 0.03 | 0.00 | 1.278 | 2.40 | 5.00 | 1.82 | 0.02 | 0 | 0 | 0 | 0.13 |
| Pb - w | 72 | 0.10 | 0.11 | 0.01 | 0.07 | 0.12 | 0.01 | 7.032 | 2.44 | 6.90 | 1.15 | 0.07 | 0.04 | 0 | 0.0645 | 0.6 |
| Zn - w | 72 | 0.30 | 0.13 | 0.01 | 0.27 | 0.33 | 0.02 | 21.44 | 0.98 | 0.68 | 0.42 | 0.10 | 0.21 | 0.1 | 0.28 | 0.65 |
| TVC - s | 72 | 300625.00 | 318847.24 | 37576.51 | 225699.57 | 375550.43 | 101664000000.00 | 2.16E+07 | 3.13 | 11.34 | 1.06 | 196597.22 | 260000 | 45000 | 200500 | 1.83E+06 |
| TC - s | 72 | 17182.08 | 21147.21 | 2492.22 | 12212.73 | 22151.43 | 447204000.00 | 1.24E+06 | 3.31 | 12.26 | 1.23 | 12232.18 | 6800 | 1710 | 11200 | 121000 |
| TS - s | 72 | 2566.81 | 5301.77 | 624.82 | 1320.95 | 3812.66 | 28108700.00 | 184810 | 4.46 | 19.86 | 2.07 | 2395.32 | 1600 | 190 | 1220 | 31000 |
| FC - s | 72 | 1458.75 | 1327.36 | 156.43 | 1146.84 | 1770.66 | 1761890.00 | 105030 | 2.15 | 5.42 | 0.91 | 908.65 | 1400 | 140 | 1125 | 6900 |
| FS - s | 72 | 197.08 | 370.20 | 43.63 | 110.09 | 284.08 | 137049.12 | 14190 | 4.47 | 20.04 | 1.88 | 164.19 | 90 | 20 | 105 | 2200 |
| VLO - s | 72 | 2589.58 | 1997.59 | 235.42 | 2120.17 | 3058.99 | 3990370.00 | 186450 | 2.29 | 6.37 | 0.77 | 1315.57 | 2200 | 270 | 2200 | 11000 |
| VC - s | 72 | 2142.92 | 1585.31 | 186.83 | 1770.39 | 2515.45 | 2513220.00 | 154290 | 2.16 | 5.66 | 0.74 | 1055.12 | 1800 | 220 | 1800 | 8700 |
| PA - s | 72 | 81.53 | 59.00 | 6.95 | 67.66 | 95.39 | 3480.73 | 5870 | 0.22 | -1.01 | 0.72 | 49.75 | 0 | 0 | 80 | 210 |
| Cd - s | 72 | 0.95 | 0.34 | 0.04 | 0.87 | 1.03 | 0.11 | 68.65 | -0.10 | -0.56 | 0.35 | 0.28 | 0.72 | 0.23 | 0.97 | 1.7 |
| Cr - s | 72 | 1.18 | 0.68 | 0.08 | 1.02 | 1.34 | 0.47 | 84.89 | 1.31 | 1.78 | 0.58 | 0.52 | 0.62 | 0.24 | 0.965 | 3.38 |
| Cu - s | 72 | 4.91 | 19.64 | 2.31 | 0.30 | 9.53 | 385.61 | 353.73 | 8.45 | 71.59 | 4.00 | 4.57 | 1.8 | 0.99 | 2.325 | 169 |
| Fe - s | 72 | 6114.38 | 3433.29 | 404.62 | 5307.59 | 6921.16 | 11787500.00 | 440235 | 0.87 | 0.24 | 0.56 | 2748.92 | 9960 | 1644 | 5371.5 | 16220 |
| Ni - s | 72 | 1.64 | 1.08 | 0.13 | 1.38 | 1.89 | 1.17 | 117.77 | 2.71 | 13.66 | 0.66 | 0.75 | 0.68 | 0.2 | 1.45 | 7.78 |
| Pb - s | 72 | 2.50 | 0.97 | 0.11 | 2.27 | 2.73 | 0.94 | 179.79 | -0.15 | -0.80 | 0.39 | 0.81 | 1.65 | 0.69 | 2.66 | 4.64 |
| Zn - s | 72 | 16.48 | 6.92 | 0.81 | 14.85 | 18.10 | 47.82 | 1186.28 | 0.25 | 0.05 | 0.42 | 5.41 | 12.45 | 2.83 | 15.87 | 34.55 |

W – Water; S – Sediment

SD - Standard Deviation; SE – Standard Error of mean; L-95% - Lower 95% CI of Mean; U-95% - Upper 95% CI of Mean; CV - Coefficient of Variation;

MAD - Mean absolute Deviation; Min – Minimum; Med – Median; Max - Maximum
